# Population ageing and sustainability of healthcare financing in China

**DOI:** 10.1186/s12962-023-00505-0

**Published:** 2023-12-19

**Authors:** Wenqing Wu, Shujie Long, Arcadio A. Cerda, Leidy Y. Garcia, Mihajlo Jakovljevic

**Affiliations:** 1 College of Management and Economics, Tianjin University, Tianjin, China; 2 Faculty of Economics and Business, University of Talca, Talca, Chile; 3 Institute of Advanced Manufacturing Technologies, Peter the Great St. Petersburg Polytechnic University, St. Petersburg, Russia; 4 Institute of Comparative Economic Studies, Hosei University, Tokyo, Japan; 5 Department of Global Health Economics and Policy, University of Kragujevac, Kragujevac, Serbia

**Keywords:** Population ageing, Medical insurance, Health financing, Sustainability, China, Elderly, Demography

## Abstract

**Background:**

In China, the healthcare financing structure involves multiple parties, including the government, society and individuals. Medicare Fund is an important way for the Government and society to reduce the burden of individual medical costs. However, with the aging of the population, the demand of Medicare Fund is increasing. Therefore, it is necessary to explore the sustainability of the healthcare financing structure in the context of population ageing.

**Objective:**

The purpose of this paper is to organize the characteristics of population ageing as well as healthcare financing in China. On this basis, it analyzes the impact mechanism of population ageing on healthcare financing and the sustainability of existing healthcare financing.

**Methods:**

This paper mainly adopts the method of literature research and inductive summarization. Extracting data from Health Statistics Yearbook of China and Labor and Social Security Statistics Yearbook of China. Collected about 60 pieces of relevant literature at home and abroad.

**Results:**

China has already entered a deeply ageing society. Unlike developed countries in the world, China's population ageing has distinctive feature of ageing before being rich. A healthcare financing scheme established by China, composing of the government, society, and individuals, is reasonable. However, under the pressure of population ageing, China's current healthcare financing scheme will face enormous challenges. Scholars are generally pessimistic about the sustainability of China's healthcare financing scheme.

**Conclusions:**

Population ageing will increase the expenditure and reduce the income of the Medicare Fund. This will further affect the sustainability of the healthcare financing structure. As a consequence, the state should pay particular attention to this issue and take action to ensure that the Fund continues to operate steadily.

## Background

Today, China has the world's second largest overall population [1], but even more important it has the greatest proportion of elderly people. Elderly groups have a greater need for health care resources than younger groups. Basic Medical Insurance is a social insurance system established in China, which is an important part of healthcare financing and helps to reduce the financial burden of individuals. Against the backdrop of population ageing, there is a growing demand for healthcare services. The issue has grown in importance in light of recent economic and social development. Some of these vulnerable bottleneck efficiency issues were further aggravated with the difficult challenges created by the corona pandemics and consecutive health policies [2]. So, can the current healthcare financing withstand the pressure of population ageing?

One major issue in early population ageing research concerned the rate of population growth, population structure, etc. Scholars have long debated the income and expenditure of Medicare Fund. In the literature on health expenditures, the relative importance of population ageing is debated. However, there are fewer studies that combine population ageing and healthcare financing.

This paper is aim to the impact mechanism of population ageing on healthcare financing and the sustainability of existing healthcare financing. Firstly, the article points out the characteristics of China's population ageing, implying that the population ageing crisis facing China is more severe. Secondly, it analyzes the rationality of the healthcare financing model with China's actual health expenditure data. Thirdly, the article subdivides the impact of population ageing on Medicare Fund revenues and expenditures and discusses the sustainability of the fund operation. Finally, summarize policies conducive to mitigating the crisis in Medicare Fund in the context of an ageing population, taking into account the actual situation in China.

## Population ageing of China

In 1982, the First World Assembly on Ageing was held in Vienna to address a range of issues faced by developed countries in the context of population ageing. By the twenty-first century, population ageing has become a globalized phenomenon and a common challenge to developed and developing countries.

According to the international standard of ageing society, China's fifth census in 2000 showed that the percentage of people aged 60 and above exceeded 10%, and the proportion of people aged 65 and above exceeded 7%. These two indicators could come to the conclusion that China has entered into an ageing society since the end of 1999 [3]. And it has been more than 20 years since then.

The population ageing of China has the following characteristics: large scale, fast growth rate, obvious regional differences, and ageing before being rich.

First, as is presented in Table 1, the population of elderly people (aged 65 years and above) in China has exceeded 200 million, ranking first in the world by the end of 2021. And scholars predicted that the number will reach a peak of 487 million in 2053 [4]. But China's ageing level (the proportion of the population aged 65 and above) is only 14.19%, slightly above the world average. This is because China has a enormous population base, this indicator does not seem severe. Currently, Japan has the highest level of ageing (28.8%) [5]. According to the World Population Prospects 2019 released by World Nations, China's ageing level will continue to deepen, reaching 26.1% by 2050 and 30.1% by 2075 [6, 7].

**Table 1:** China's population by age group, 2014–2021

Indicators/million	2014	2015	2016	2017	2018	2019	2020	2021
Total	1367.82	1374.62	1382.71	1390.08	1395.38	1400.05	1411.78	1412.6
0–15	239.57	241.66	244.38	247.19	248.6	249.77	253.38	263.02
16–59	915.83	910.96	907.47	901.99	897.29	896.4	894.38	882.22
Over 60	212.42	222	230.86	240.9	249.49	253.88	264.02	267.36
Over 65	137.55	143.86	150.03	158.31	166.58	176.03	190.64	200.56

Data source: Statistical bulletin on the economic and social development of the population

**Fig. 1:**
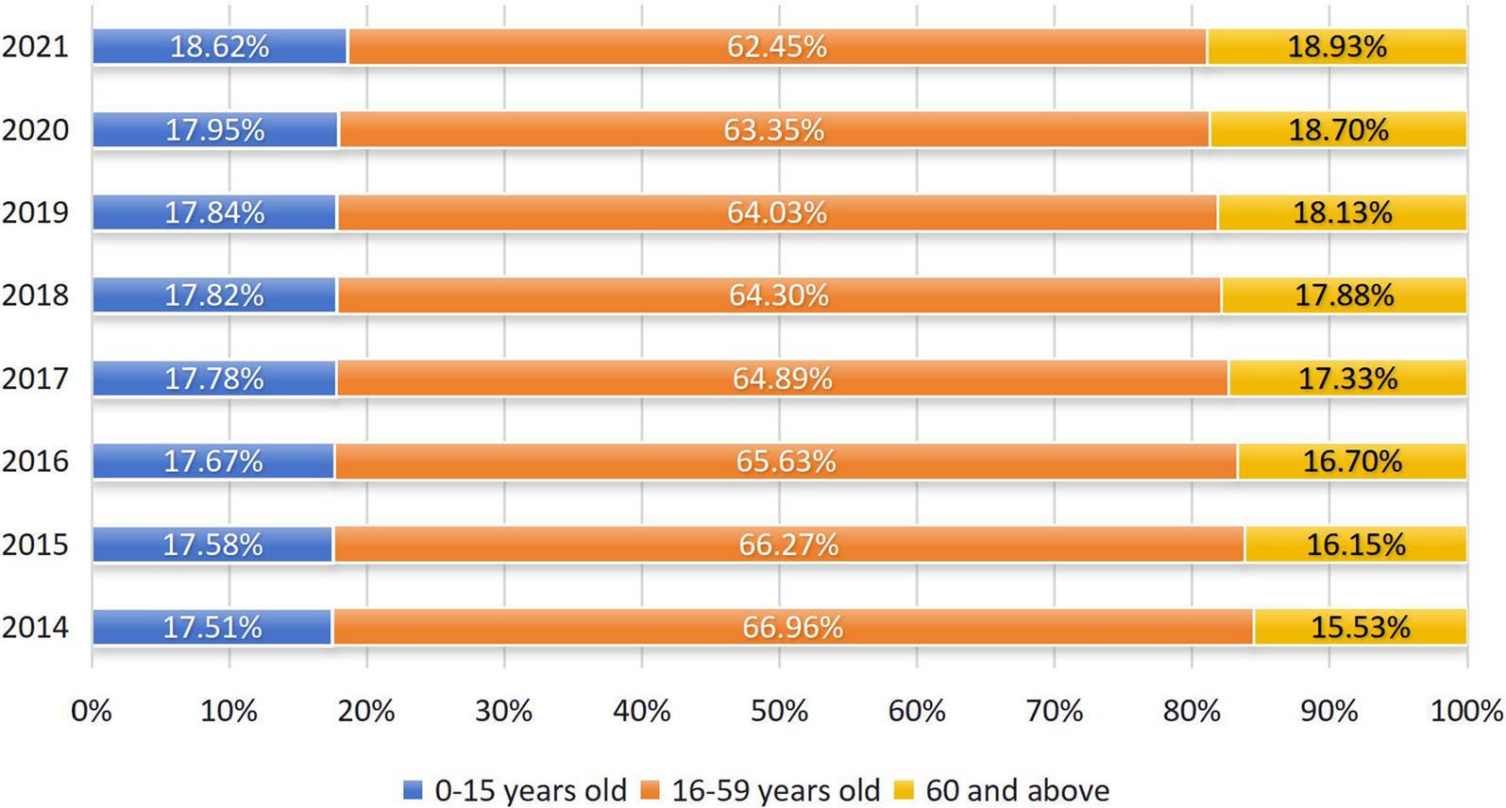
China’s population age scheme (million persons), 2014–2021. Data source: Statistical bulletin on the economic and social development of the population

Second, China's population is ageing much faster than of other ageing countries. As can be seen from Table 1, the number China's elderly population has continued to increase in recent years. As shown in Fig. 1**,** the proportion of people aged 60 and above increases by one percentage point every two years. By the end of 2021, the proportion has exceeded 18%, indicating China has entered a deeply ageing society. Compared with developed countries such as France, the UK, and Germany, China takes the shortest time from entering ageing to deeply ageing.

Third, the ageing of the population is not identical in different geographic regions of China. There are disparities between urban and rural areas [8]. The proportion of elderly population in rural areas is higher than that in urban areas, as well as the rate of ageing [9]. For one reason, the average life expectancy of the population in urban areas is 5–6 years longer than that in rural areas due to good physical and medical conditions [10]. And then, the fertility rate in urban areas is lower than that in rural areas. Last but not the least, the migration of the labor force population from rural to urban areas decreases the base of rural population, intensifies the process of rural ageing [11].

Fourth, when China entered the ageing process in 2000, the GDP per capita was just above $1000. However, developed economies such as Sweden, Japan, the UK, and Germany, had a GDP per capita of about $5000–10,000 at that time, indicating that these countries had sufficient financial resources to solve the problem of medical security for the elderly. China was therefore, dealing with problems at a low level of guaranteed income and social support, which make it unable to afford the issue brought on by the population's rapid ageing [12]. China is still a developing country and is at the primary stage of socialism. These demographic changes lead to a mismatch between the ageing of China’s population and the nation's ability to afford healthcare services. Similar wider patterns of third demographic transition were visible throughout an array of diverse LMICs nations of the Global South in decades following the Cold War era [13].

In a word, population ageing has become an issue that cannot be ignored in China. The huge elderly population will result in huge healthcare expenditures. The difference between the development of socio-economic level and the speed of ageing will create a gap. So, what is the current financial structure of healthcare in China?

## Healthcare financing in China

In the World Health Organization’s World Health Report, healthcare financing has three functions, raising funds, sharing risks, and purchasing services. Elderly people have a higher need for healthcare than younger people, so rapidly population ageing places higher demands on the country’s healthcare system and funding scheme.

### Total health expenditures

The total health expenditures (THE) is the total amount of money invested in a country or region over a certain period of time to carry out health service activities. It reflects the degree of importance attached to healthcare and the level of cost burden of the whole society under certain economic conditions. At the same time, it can also reflect the main features of the health financing model and the fairness and rationality of health financing. As shown in Table 2, the scale of THE in China has increased year by year, from 1998.04 billion yuan in 2010 to 6584.14 billion yuan in 2019.

**Table 2:** Total health expenditures in China, 2010–2019

Year	THE/billion yuan	per capita THE /yuan	THE%GDP
2010	1998.04	1490.1	4.85
2011	2434.59	1807	4.99
2012	2811.90	2076.7	5.22
2013	3166.90	2327.4	5.34
2014	3531.24	2581.7	5.49
2015	4097.46	2980.8	5.95
2016	4634.49	3351.7	6.21
2017	5259.83	3783.8	6.32
2018	5912.19	4237	6.43
2019	6584.14	4702.8	6.64

Data source: China Health Statistical Yearbook

The proportion of total health expenditures to GDP is usually used to reflect the strength of the State's investment in health during a certain period of time. If this ratio is too low, it indicates that the state and society do not attach enough importance to the health of the people. If the ratio is too high, it exceeds the level that society can afford, and will inevitably increase the burden of health expenditures on the government, society and individuals [14].

**Fig. 2:**
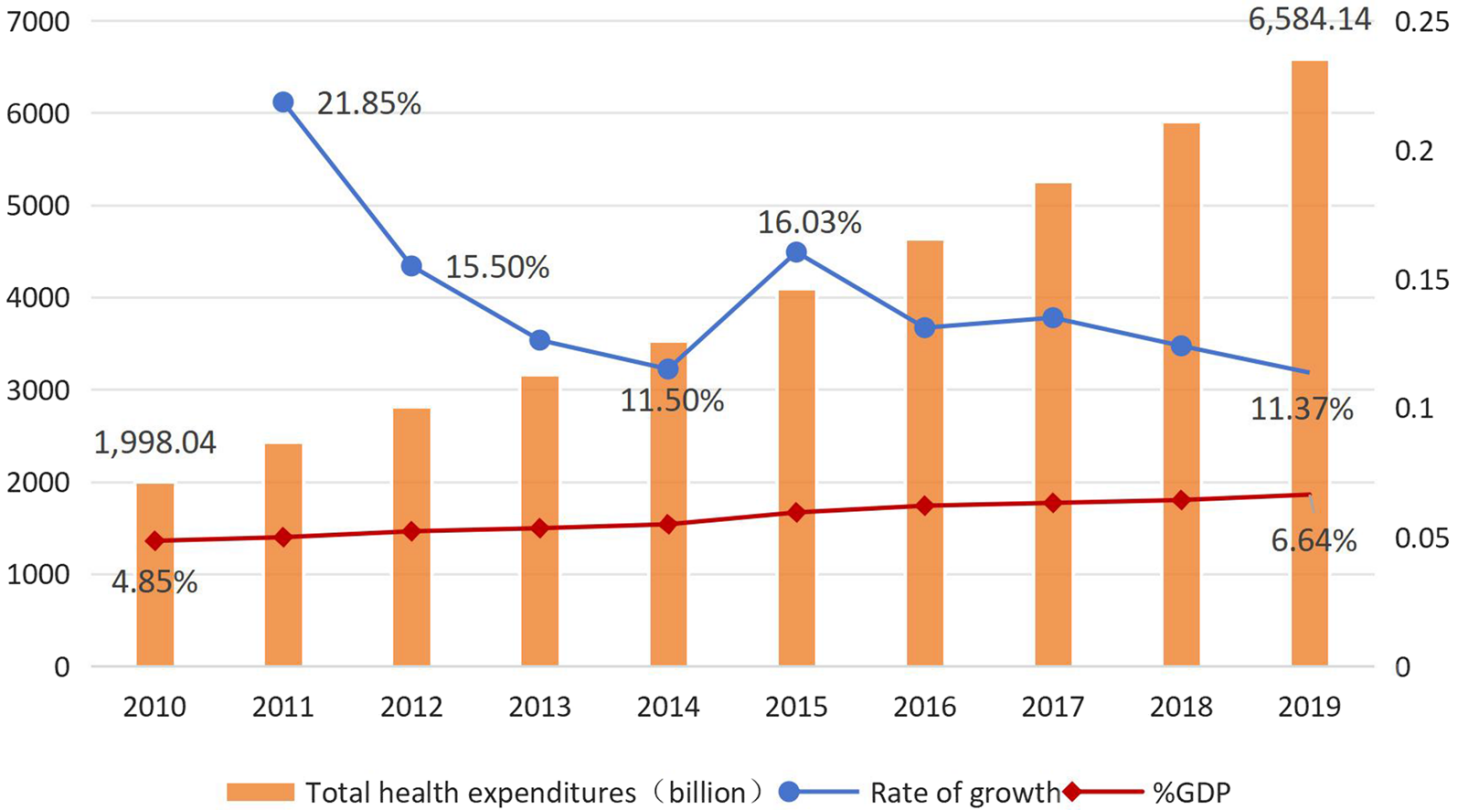
Total Health Expenditures in China.2010–2019. Data source: China Health Statistical Yearbook

In China, the proportion of THE of GDP increased from 4.85% in 2010 to 6.64% in 2019. But the growth rate decreased from 21.85% in 2011 to 11.37% in 2019. Wang et al. [15] stated that China’s appropriate ratio of total health costs of GDP is 5.5% [15].As shown in Fig. 2, the percentage of GDP continued to rise between 2010 and 2019 and reached 6.64% in 2019, indicating a significant increase in demand for healthcare among residents. The per capita health expenditures will keep growing and is expected to reach 14,129.2162 yuan in 2030, which is nearly three times higher than in 2019. The growth rate was maintained at approximately 10% with an overall decreasing trend [16]. However, China's THE is still low compared to international levels and well below that of high-income countries (8% of GDP for THE) [17].

Many scholars have found that THE is positively related to the economic level. Wang and Zhang [19] proposed that the growth rate of THE must be coordinated with the pace of national economic development [18]. From 2010 to 2019, China's per capita health expenditures increased from 1490.1 yuan to 4702.8 yuan, an increase of 3.16 times, which far exceeded the economic development in the same period (per capita GDP increased from 30,808 yuan to 70,892 yuan, an increase of 2.30 times). The growth of the two does not match, and the per capita health expenditures is growing too fast, which will aggravate the problem of “difficult and expensive to get healthcare service”. Wang et al. [18] confirmed this by constructing a GM (1,1) prediction model [18].

The significant regional differences in health expenditures are accompanied by inequity in socioeconomic development in China [19]. As shown in Table 3, health expenditures in urban areas was three times higher than that in rural areas. There is a large gap in healthcare consumption in the long run. In the context of population ageing, healthcare demands differ between urban and rural residents. It is found that urban ageing has a positive effect on per capita health expenditures, while rural ageing has little effect. In contrast to urban ageing, rural ageing results in a significant decline in real per capita out-of-pocket health expenditures. The reason for the difference is that the healthcare needs of the rural elderly population are not effectively met [20].

**Table 3:** Urban and rural health expenditures in China, 2010–2016 (billion yuan)

	2010	2011	2012	2013	2014	2015	2016
Urban	1550.86	1857.19	2128.05	2364.50	2657.56	3129.79	3545.80
Rural	447.18	577.40	683.85	802.40	873.68	967.68	1088.69

Data source: China Health Statistical Yearbook

Overall, China’s THE is increasing year by year, indicating that the Chinese government and its residents are gradually raising the importance of health. However, compared to other countries, the level of THE is still low [21]. Moreover, the growth rate is too fast and exceeds the level of economic development in the same period.

### Health financing scheme

In the Chinese Health Statistical Yearbook classification, according to the source of funds, total health expenditures (THE) can be categorized into government health expenditures (GHE), social health expenditures (SHE), and out-of-pocket payments (OPP).

Government health expenditures (GHE) are spendings at all levels of government for health services, medicare subsidies, health and medicare administration, and population and family planning affairs expenditures.

Social health expenditures (SHE) refer to the financial inputs made by all sectors of society to health undertakings, in addition to government expenditures. It includes social medical insurance expenditure, commercial health insurance premiums, expenditure on social medical services, social donations and assistance, and income from administrative fees.

Out-of-pocket payments (OPP) refers to the cash payments made by urban and rural residents when they receive various types of medical and health services. It refers to the expenses paid by the residents themselves when they see a doctor under the various types of health insurance systems. It can reflect the extent of the burden of medical and health costs on urban and rural residents.

Li et al. [22] proposed an appropriate composition of health financing in China: the tripartite ratio of government, society, and individuals is 4:3:3 [22]. As shown in Table 4, the current composition of health financing in China is broadly consistent with the ideal state inferred by scholars.

**Table 4:** Scheme of health financing in China, 2010–2019

Year	GHE %	SHE %	OPP %
2010	28.69	36.02	35.29
2011	30.66	34.57	34.8
2012	29.99	35.67	34.34
2013	30.1	36	33.9
2014	29.96	38.05	31.99
2015	30.45	40.29	29.27
2016	30.01	41.21	28.78
2017	28.91	42.32	28.77
2018	27.74	43.66	28.61
2019	27.36	44.27	28.36

Data source: China Health Statistical Yearbook

In a mixed public–private healthcare financing scheme, it is widely believed that the greater the share of public funding, the lower the risk of healthcare distress. WHO published World Health Statistics: 2022. According to this report, the share of government health expenditures(GHE) in THE for different types of countries is 21% for low-income countries, 34% for lower-middle-income countries, 37% for upper-middle-income countries, and 48% for high-income countries [17]. Thus, the higher the level of the economy, the larger the share of GHE.

For China, as can be seen in Table 4, the share of GHE was 27.36% in 2019. So, the indicator of China is well below the average of lower-middle-income nations, and there is a significant difference between China and high-income nations. The overall growth is volatile, indicating that the input mechanism of government spending is not stable and that the government-led role played is insufficient. Sustainability must be further optimized [23].

A critical feature of financial strain on people's health and equity in health financing is the percentage of out-of-pocket payments (OPP) [24]. According to the report published by the WHO, the proportion of OPP in low-income countries is 44% [25]. As shown in Table 4, the proportion of OPP continued to decrease from 35.29% in 2010 to 28.36% in 2019, meaning that the ability to resist risks has increased. It has achieved the goal of “reducing the proportion of OPP to about 28% in 2020”, as proposed in the “Health China 2030 Plan” [26].

In this section, we learned about the trends in China's total health expenditures in recent years. After that, we analyze the overall structure of health expenditures in China. The role played by the government and society is gradually increasing, with less and less pressure on individuals to pay. In the future, public health expenditures will gradually become the primary source of health financing [27, 28].

### Medicare fund

Medicare Fund is an essential component of healthcare financing and plays a vital role in maintaining social stability. In China, it is called Basic Medical Insurance (BMI).

In 1998, China introduced Urban Employees’ Basic Medical Insurance (UE-BMI), which gradually replaced labour insurance and socialized medicine. Since 2003, the New Rural Cooperative Medical System (NRCMS) has been working on a pilot basis, and a medical assistance system has been implemented for poverty groups. In 2007, the Urban Residents’ Basic Medical Insurance (UR-BMI) was piloted. They differ in coverage, reimbursement scope, treatment standards, and other aspects [29]. At this point, China has established a Basic Medical Insurance (BMI) system covering the urban employed population, vast rural population, urban non-employed population, and poverty groups. In 2016, the NRCMS and UR-BMI were integrated, called Urban and Rural Resident Basic Medical Insurance (URRBMI). The composition of the medical security system for residents at each stage was obtained from the National Health Service Survey in 2003, 2008, and 2013, as shown in Table 5.

**Table 5:** Residents’ participation in medical insurance in surveyed areas (%)

	2008	2013	2018
UE-BMI	12.7	21	23.4
UR-BMI	3.8	13.2	73.3
NRCMS	68.7	51.1	
Other social medical insurance	1	0.5	0.4
No social medical insurance	12.9	4.4	2.9
Socialized medicine	1		
Cooperative medical care for urban and rural residents		9.9	

Data source: China Health Statistics Yearbook, National Health Service Statistics

As shown in Table 5, in 2018, 97.1% of the residents participated in social medical insurance, and the coverage of BMI reached 96.7%. The data showed that medical policy subsidizes most Chinese citizens seeking medical care. Studies show that UR-BMI enrollees are the least likely of the three social health insurance systems to incur out-of-pocket costs [30].

China's BMI has a pay-as-you-go financing system. Therefore, the operation of the Medicare Fund can be accurately reflected by the total revenue, outlays, and year-end balances. As seen in Table 6**,** during 2013–2019, the total revenues of the Medicare Fund exceeded 2 trillion yuan in 2018, with an average growth rate of approximately 20%. At the same time, the total expenditures of the Medicare Fund also continue to increase, but the balances are maintained and show an upward trend [31]. The overall balances of the Medicare Fund are good, indicating that the supply side of the Medicare Fund is larger than the demand side during the period. And the basic medical insurance can meet the residents' demand for healthcare services in an appropriate state.

**Table 6:** China's basic Medicare Fund revenues and expenditures (billion yuan)

Year	Revenues	Expenditures	Year-end balance			
			Social Integration account	Personal account	Total	Rising %
2013	824.8	680.1	579.4	332.3	911.7	
2014	968.7	813.4	673.2	391.3	1064.5	16.76%
2015	1119.3	931.2	811.4	442.9	1254.3	17.83%
2016	1308.4	1076.7	976.5	520	1496.5	19.31%
2017	1793.2	1442.2	1323.4	615.2	1938.6	29.54%
2018	2138.4	1782.2	1615.6	728.4	2344	20.91%
2019	2442.1	2085.4	1927	1042.7	2969.7	26.69%

Data source: China Health Statistical Yearbook, National Bureau of Medical Security Statistical Bulletin

Inequality in Medicare Fund also exists in different areas. With the introduction of NRCMS and UR-BMI in China, the disparity in health financing between urban and rural inhabitants has not decreased as anticipated [32]. Urban residents have better medical coverage and pay higher medical insurance costs than rural residents [33]. The basic Medicare Funds premiums and balances vary widely in different regions. Greater fund balances and lower contribution ratios are typical in more developed provinces like Beijing, Guangdong, and Zhejiang, whereas the inverse is true for less developed provinces like Qinghai, Gansu, and Ningxia [34].

As an important way of government social security, Basic Medical Insurance has distinctive Chinese characteristics. It helps to reduce the financial burden of individuals. And it adopts the mechanism of joint contribution by enterprises and individuals, and stipulates that retirees no longer pay contributions. So, in the context of the current deepening ageing, how will healthcare spending change and will China's current healthcare financing model be sustainable?

## Population ageing and sustainability of medicare fund

### The Medicare Fund’s expenses

Data show that the per capita health expenditures for people over 65 is 7.25 times higher than those under 25, 1.61 times higher than those aged 25–59, and 3.47 times higher than those aged 60–64 [35]. So, it can be seen that elderly adults have a greater need for healthcare services than younger adults because of deteriorating physical function and reduced immunity.

It should be realized that population ageing has a positive effect on the growth of total health expenditures(THE). However, some scholars have suggested that population ageing has little effect on THE. In a study, 21.2% of the THE growth was driven by population ageing [36]. Wang et al. (2017) verified the weak effect of increased ageing on out-of-pocket payments through a vector autoregressive model [37]. In addition, it is influenced by other factors like the develpoment of economic levels.

Using a B-VAR model by Lopreite and Mauro, THE in Italy responds more to the ageing index than to life expectancy and economic growth [38, 39], and so does China [40]. Liu et al. (2022) found that economic factors such as GDP per capita and the proportion of the tertiary sector to GDP significantly affected THE [23]. Amiri and Linden [41] conducted a two-way causality study of THE and economic growth and found a broadly dominant bilateral relationship between GDP and THE [41]. Guo et al. [16] developed a VAR model between the THE and influencing factors, and the results showed that the level of urbanization had the most significant effect. The effect of population ageing is not apparent at present, but it will have a long-term positive impact on the THE in China [16].

Studies show that the deepening ageing of the population will lead to a rapid increase in Medicare Fund expenses in China [42]. The per capita expenses of the Medicare Fund for retirees was much higher than that of active participating workers. On the one hand, expansion of basic health insurance coverage has expanded the population of insured persons. On the other hand, scientific and technological advances in the field of health care have prolonged the life expectancy of the elderly. Both of them increase the use of the Medicare Fund.

Therefore, combining the two main factors of population ageing and the level of economic development, China's total health expenditure will continue to rise. The Medicare Fund, as an important means of payment, will be under tremendous pressure to pay.

### The medicare fund’s revenues

Ageing populations result in a declining young labour force and an increasing population of senior residents, creating a problem for fund revenues [43]. In China's UE-BMI, retired participants and their former units no longer pay medical insurance premiums. The insurance fees paid by active employees are used for their own medical treatment, in addition to retirees’ medical expenses. Therefore, the higher the proportion of retirees, the greater the pressure on the UE-BMI [44]. When the contribution ratio of the Medicare Fund is fixed, a decrease in the number of working laborers will lead to a reduction in the supply of funds. The health system built on the demographic growth model will not be able to cope with the costs of long-term care and medical expenditures associated with ageing [43]. Consequently, population ageing is an essential factor affecting the sustainability of the Medicare Fund. At the same time, Chinese finance is insufficiently resilient to population ageing, and financial assistance is scarce [45], which increases the strain on medical spending.

During the 10 years between 2009 and 2019, the number of insured workers in UE-BMI increased from 219.37 million to 316.81 million, of which the number of insured retirees rose from 55.27 million to 83.73 million. The change in the number of insured workers versus insured retirees represents a change in the retirement-to-work ratio, which increased from 33.68 percent to 35.92 percent. The ageing of the population has led to changes in the age scheme of medical insurance participants.

Among the people covered by the system, the number of contributors is decreasing, and the number of beneficiaries is increasing, a trend we call “system ageing”. Sun and Chi established a multidisciplinary model to measure the “systematic ageing risk” of the urban employee health insurance system. They found that an ageing population will cause the fund to face a serious financial crisis [46]. In the long term, the “system ageing” will continue to increase, threatening the stability and sustainability of the medical insurance system.

Qiu et al. [47] discovered that the Medicare Fund’s revenues and expenses increased in parallel in China from 2008 to 2017. At the same time, the balance kept growing but at a very slow rate, indicating that it hides a significant risk [47]. However, Jia and Zhao [48] find that the increase in per capita fund expenditures is greater than the increase in per capita fund revenues for employee health insurance, resulting in a risk to the sustainability of the fund [48].

Some scholars have made model predictions for the break-even situation of medical funds, and their results are primarily negative. Zeng and Yang [44] examined the effects of population ageing on Medicare Fund spending using a system dynamics approach. The results showed that Medicare Fund balances are decreasing yearly and will be in deficit by 2025 [44]. In the early stages, central and local financial subsidies cover the funding gap. When the funding gap reaches 41.11% of the revenues of the social coordination fund in 2050, the repayment problem of the Medicare Fund will not be neglected [34]. Ge and Wang [49] construct a dynamic break-even prediction model to obtain the break-even situation of the Medicare Fund in China from 2020 to 2035. The results show that the operation of the Medicare Fund is not sustainable. The current year's balance is expected to show a shortfall for the first time around 2026, and the accumulated balance is expected to show a shortfall around 2034 [49]. Sun and Zhao [50] used the balance rate of integrated funds as a proxy variable to measure the sustainability of the basic medical insurance system. The higher the balance rate is, the more sustainable it is. The result showed that the retirement-to-employment ratio kept a continuously rising trend after 2011, and “system ageing” became more serious, which caused a decrease in the coordinated fund balances [50]. It is important to emphasize that the underlying long term trends and hidden patterns of fiscal flows within the Chinese health sector are largely comparable to similar unfolding development among other leading Emerging BRICS markets [51]. These large nations continue to lead in terms of real GDP growth worldwide [52]. Consequence is the huge impact to global supply and demand of many essential pharmaceutical, medical devices and services particularly in terms of South-South trade with the vast majority of Belt and Road countries [53].

Overall, Basic Medical Insurance(BMI) adopts the mechanism of joint contribution by enterprises and individuals, and stipulates that retirees no longer pay contributions. So, all the revenues of the Fund come from active employees. Ageing has increased the ratio of retired-active workers year by year, creating a relatively minor contributor group. This has a negative impact on the solvency of Medicare Fund [14]. Additionally, there has been a large rise in healthcare spending due to the rising emphasis on healthy life and the enormous need for healthcare services from the ageing population. It is undeniable that China's BMI system has faced with the financial dilemma of decreasing revenues and increasing expenses.

## Government policies

In the face of the inevitable trend of population ageing, and drawing on the precedents of other countries, China is trying to explore the experiment.

Firstly, raising the statutory retirement age. Raising the statutory retirement age is a typical international approach for addressing the crisis of an ageing population. By sorting out the retirement age situation in 170 countries or regions around the world, it was found that developed countries generally implemented a retirement age of 65 or older [54]. Some developing countries with a per capita GDP level similar to China generally have an older retirement age, so China's statutory retirement age is still very low. Therefore, it makes sense to explore a flexible retirement system by gradually increasing the retirement age [55].

At the end of 2015, China studied and proposed a gradual delay in retirement age policy. In 2021, the “14th Five-Year Plan” clearly suggested "gradually delay the statutory retirement age". As of September 2021, 21 provinces, including Beijing, Hunan, and Jiangxi, have completed the consultation process. In January 2022, the Human Resources and Social Security Department of Shandong Province issued a document that landed the first nationwide delayed retirement policy.

Secondly, increase the fertility rate. Since the implementation of the family planning policy, China's population growth has slowed, and the pressure on resources and the environment has eased. To improve China’s population scheme and actively cope with ageing, China has gradually adjusted its fertility policy. The "two-child policy" was fully implemented in 2015, and the "three-child policy" was launched in 2021. However, some scholars have found that increasing the fertility rate does not effectively improve the difference in revenues and expenditures of BMI, through simulated policy experiments with different fertility rates [56].

Thirdly, extending the contribution period. Most scholars believe that although population ageing is the direct cause of "system ageing", the mechanism of non-contribution by retirees is an important institutional trigger [50]. Participating retirees pay a certain amount of medical insurance premiums, which can alleviate pressure on the fund and enhance the awareness of saving among retirees.

In 2022, Shandong and Guangdong issued a document stating that the contribution years for employee health insurance should be increased. It also stipulates that the minimum contribution period for male insured workers is 30 years and 25 years for females, and that the contribution period for employees will increase year by year. The extension of the contribution period will increase the contribution of the insured contributors to the Medicare Fund and strengthen the Fund’s ability to operate. In addition, Zhang Yinghua, executive researcher of the World Social Security Research Center of the Chinese Academy of Social Sciences, analyzed that the unification of the minimum contribution period is conducive to speeding up the pace of provincial coordination of basic medical insurance and solving the problem of differences in the contribution burden of participants between regions.

Academic researchers have also discussed policy improvements [57]. First, the revenues of the medical insurance coordination fund come mainly from enterprise contributions. Therefore, increasing the enterprise contribution rate is conducive to balancing account [34]. Second, establishing a multi-pillar medical security system of government, the market, and society, such as the combination of basic medical insurance and commercial insurance, can improve the risk resistance of the Medicare Fund [47, 55]. Third, other scholars suggested replacing hospitalization with long-term care, and analysis shows that people's investment in longevity insurance reduces health insurance spending [58]. Compared with other countries, China's legal system for the older lags behind the progress of population ageing in terms of development speed and quantity. Therefore, the country should learn from good practices and accelerate the pace of improving the legal system for the elderly [4]. However, if we enter deep ageing, it will be difficult to alleviate the shortage of fund supply through policies [50]. While broadening the revenues of medical funds, there is also a need to strengthen medical cost monitoring and control the increase in medical costs [59].

## Conclusion

As China gradually enters a deeply ageing society, the influence of an ageing population on the Medicare Fund is further amplified. On the one hand, labour forces population decreases, resulting in a continuous decrease in the proportion of contributors to insured people. On the other hand, ageing population creates a huge demand for healthcare services. All in all, the ageing population poses a great threat to the balance of Medicare Fund, making it difficult to ensure sustainable development.

By combing through a large amount of literature, on the one hand, this paper takes population ageing and healthcare financing as the theme, and gives a detailed overview of the current situation and characteristics of both. On the other hand, this paper theoretically elucidates the mechanism of population ageing on healthcare financing. Based on the above analysis, this paper organizes the relevant policies and suggestions from the practical point of view, with a view to having certain reference value for practical work.

## Data Availability

The datasets analyzed during the current study are available in China Bureau of Statistics.

## Abbreviations

GDP: Gross Domestic Product
LMICs: Lower middle income countries
THE: Total health expenditures
GHE: Government health expenditures
SHE: Social health expenditures
OPP: Out-of-pocket payments
BMI: Basic Medical Insurance
UE-BMI: Urban Employees’ Basic Medical Insurance
NRCMS: New Rural Cooperative Medical System
UR-BMI: Urban Residents’ Basic Medical Insurance
URRBMI: Urban and Rural Resident Basic Medical Insurance
